# Bailout MitraClip therapy for deteriorated systolic anterior motion–related severe mitral regurgitation post-alcohol septal ablation: a case report

**DOI:** 10.1093/ehjcr/ytad599

**Published:** 2023-11-27

**Authors:** Shinsuke Nakano, Hiroyuki Yamamoto, Nobuyuki Takahashi, Tomofumi Takaya

**Affiliations:** Division of Cardiovascular Medicine, Department of Internal Medicine, Hyogo Prefectural Harima-Himeji General Medical Center, 3-264 Kamiya-cho, Himeji 670-8560, Japan; Division of Cardiovascular Medicine, Department of Internal Medicine, Hyogo Prefectural Harima-Himeji General Medical Center, 3-264 Kamiya-cho, Himeji 670-8560, Japan; Division of Cardiovascular Medicine, Department of Internal Medicine, Hyogo Prefectural Harima-Himeji General Medical Center, 3-264 Kamiya-cho, Himeji 670-8560, Japan; Division of Cardiovascular Medicine, Department of Internal Medicine, Hyogo Prefectural Harima-Himeji General Medical Center, 3-264 Kamiya-cho, Himeji 670-8560, Japan; Department of Exploratory and Advanced Search in Cardiology, Kobe University Graduate School of Medicine, Kobe, Japan

**Keywords:** Alcohol septal ablation, Case report, Complication, Left ventricular outflow tract obstruction, Mitral regurgitation, MitraClip

## Abstract

**Background:**

Percutaneous alcohol septal ablation (ASA) is a non-surgical treatment for symptomatic hypertrophic obstructive cardiomyopathy. It has a potential risk for systolic anterior motion (SAM)–related mitral regurgitation (MR) deterioration, leading to acute congestive heart failure. In such clinical scenarios, additional surgical interventions for SAM-MR are risky.

**Case summary:**

A 70-year-old man experienced acutely deteriorated heart failure caused by SAM-related MR following ASA, for which venous-arterial extracorporeal membrane oxygenation (ECMO) and a percutaneous left ventricular assist device (Impella CP, Abiomed, MA, USA) were required. Transoesophageal echocardiography showed that an interventricular septal oedematous protrusion led to a large coaptation gap of mitral leaflets with a pseudo-prolapse of the posterior mitral leaflet (PML). Because of his prohibitive surgical risks, we opted for transcatheter edge-to-edge mitral valve repair with MitraClip therapy. After removing the Impella device, an XT clip (Abbott Vascular, CA, USA) was located to cover the pseudo-prolapsed PML, resulting in optimal MR reduction with an acceptable mean transmitral valve-pressure gradient. Thereafter, his heart failure was well controlled, and venous-arterial ECMO was successfully removed on post-MitraClip Day 2.

**Discussion:**

This case demonstrated that MitraClip therapy rescued the patient from a rare complication of severe acute heart failure with haemodynamic collapse caused by massive SAM-related MR following ASA. MitraClip therapy can be a feasible, less-invasive interventional therapy for SAM-related MR in cases with acceptable severity of iatrogenic mitral stenosis post-MitraClip implantation.

**Learning points**

- Percutaneous alcohol septal ablation can lead to an interventricular septal oedematous protrusion, potentially causing refractory systolic anterior motion (SAM)–related mitral regurgitation (MR).
- Transcatheter edge-to-edge mitral valve repair with MitraClip therapy can be a feasible, less-invasive interventional therapy for SAM-related MR.

## Introduction

Percutaneous alcohol septal ablation (ASA), a non-surgical septal reduction therapy, is effective in decreasing left ventricular outflow tract (LVOT) obstruction and ameliorating systolic anterior motion (SAM)–related mitral regurgitation (MR) in patients with symptomatic hypertrophic obstructive cardiomyopathy (HOCM).^1^ Alcohol septal ablation–related complications are known, including unintended remote myocardial infarction, malignant ventricular arrhythmia (ventricular fibrillation, 2.2%), pacemaker requirement for atrio-ventricular block (10.5%), and sudden death (1.9%); however, deteriorating SAM-related MR following ASA is rare.^2^

Transcatheter edge-to-edge mitral valve repair (M-TEER) with MitraClip therapy (Abbott, IL, USA) effectively improves cardiac outcomes in patients with chronic heart failure caused by primary (degenerative) or secondary (functional) severe MR.^3,4^ Several cases have been reported to the effect that M-TEER was useful for high surgical-risk patients with acute heart failure caused by SAM-related MR.^5,6^ However, the efficacy of M-TEER for deteriorated SAM-related MR following ASA has not been explored.

Herein, we present a rare case of SAM-related MR following ASA that was rescued by M-TEER with MitraClip therapy.

## Summary figure

**Figure:**
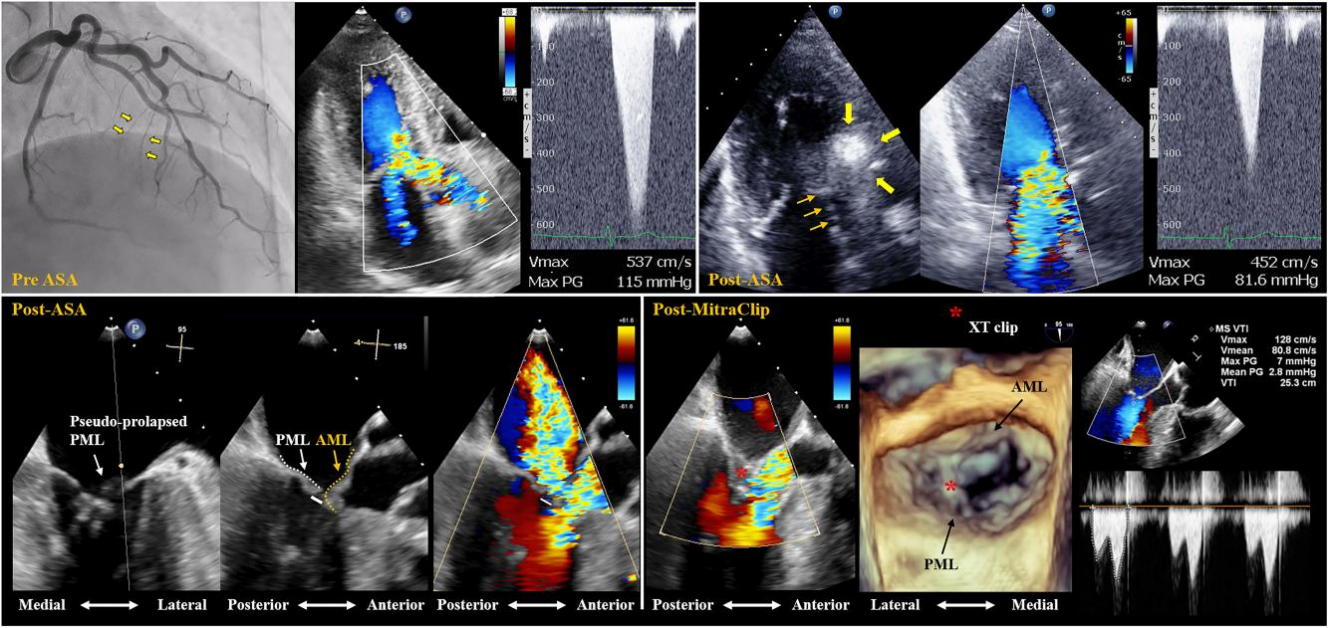


## Case presentation

A 70-year-old man, diagnosed with HOCM 10 years prior, presented with worsening exertional dyspnoea. He was taking several medications for HOCM, including beta-blocker (bisoprolol 5 mg/day), calcium channel blocker (diltiazem 100 mg/day), and cibenzoline (100 mg/day), under which the mean LVOT pressure gradient (PG) was well controlled to <30 mmHg. However, his symptoms gradually worsened to New York Heart Association functional Class 3.

**Figure 1:**
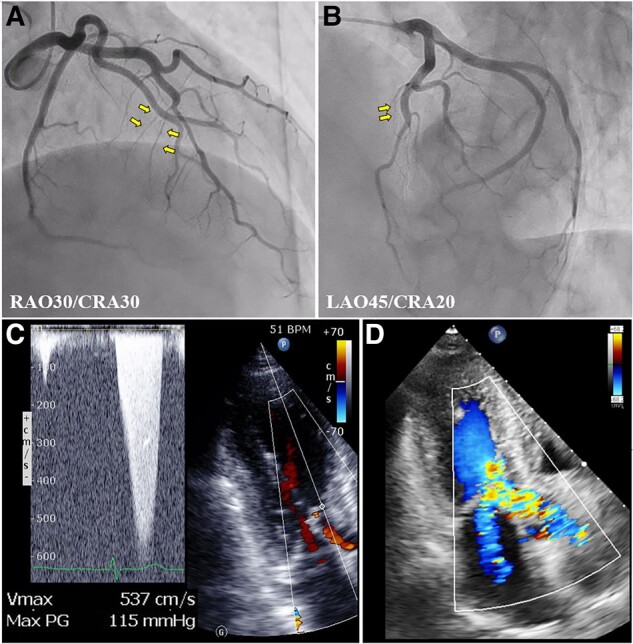
Pre-operative evaluation of the left ventricular outflow tract obstruction and systolic anterior motion–related mitral regurgitation. (*A* and *B*) Initial coronary angiogram shows target septal branches (arrows). (*C* and *D*) Transthoracic echocardiography showing a left ventricular outflow tract pressure gradient and mild systolic anterior motion–related mitral regurgitation before percutaneous alcohol septal ablation. CRA, cranial; LAO, left anterior oblique; RAO, right anterior oblique.

On admission, physical examination showed no abnormalities except systolic cardiac murmur with Levine IV/VI. He was haemodynamically stable (blood pressure: 98/66 mmHg; heart rate: 52 b.p.m.). Laboratory test results showed a significantly elevated brain natriuretic peptide level (797 pg/mL) and moderate renal dysfunction (estimated glomerular filtration rate, 44 mL/min/1.73 m^2^). Transthoracic echocardiography showed a normal ejection fraction of 69.0%, severe LVOT obstruction that was deteriorated to a maximum (mean) LVOT PG of 115 (52) mmHg, and mild SAM-related MR. Coronary angiography clearly showed several septal perforators feeding into a hypertrophic septum (*Figure 1A–D*). After the patient was informed in detail about the pros and cons of both surgical myectomy vs. ASA as treatment options, he refused treatment with surgical myectomy and requested ASA. The heart team agreed with the patient’s choice of ASA for reducing the LVOT obstruction.

**Figure 2:**
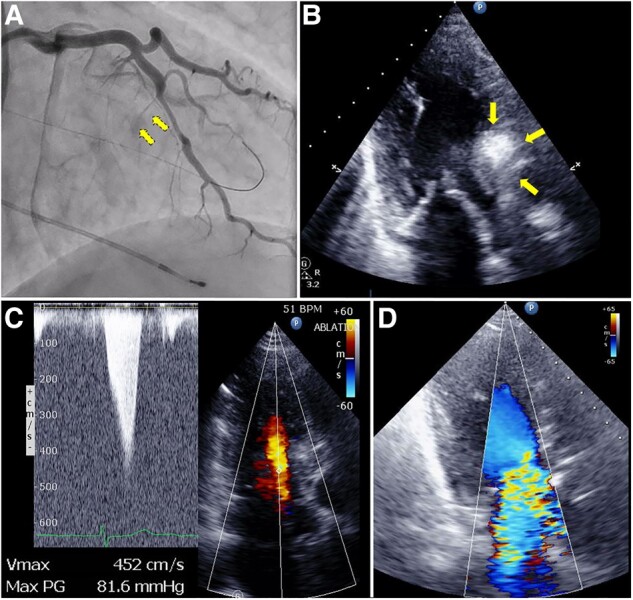
Post-alcohol septal ablation. (*A*) Coronary angiography after alcohol septal ablation shows an obstruction of septal perforators. (*B*) Interventricular septal oedematous protrusion (yellow arrows) can be observed, (*C*) with insufficient left ventricular outflow tract pressure gradient reduction, and (*D*) deteriorated systolic anterior motion–related mitral regurgitation after alcohol septal ablation.

**Figure 3:**
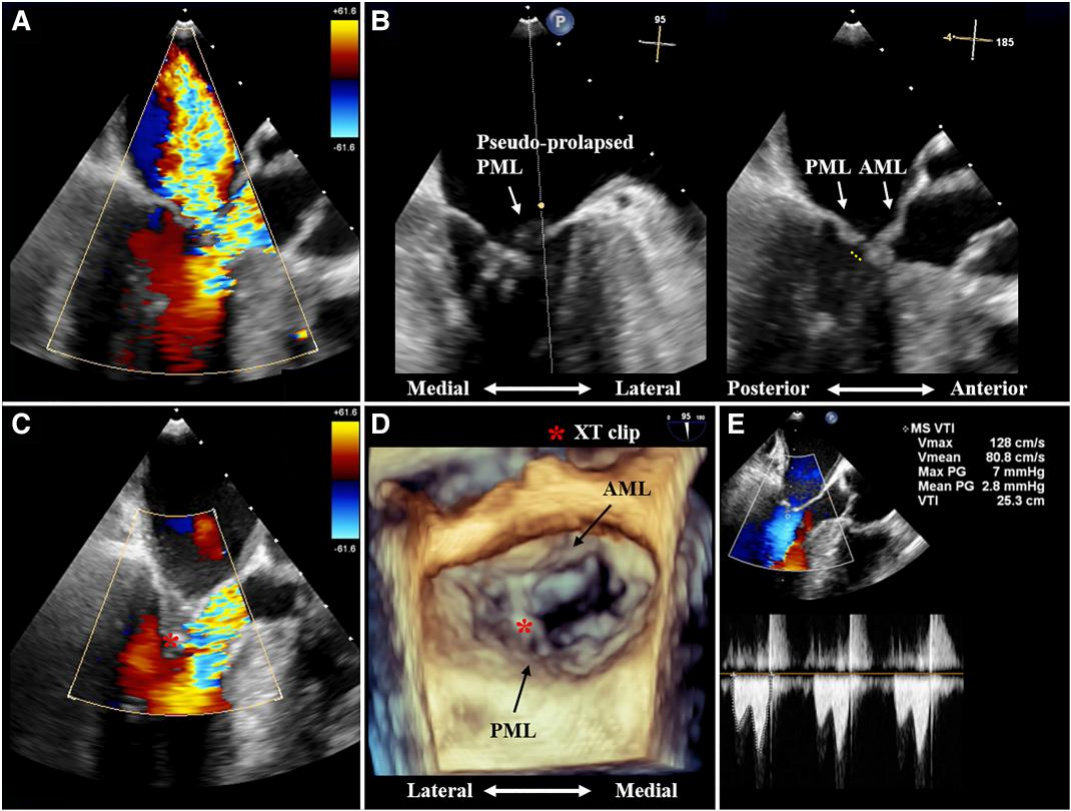
Transoesophageal echocardiography evaluation during MitraClip therapy. (*A* and *B*) Transoesophageal echocardiography shows that the massive systolic anterior motion–related mitral regurgitation is caused by a newly developed large mitral leaflet coaptation gap with a pseudo-prolapse of the posterior mitral leaflet. The white line denotes the coaptation gap. (*C–E*) Transoesophageal echocardiography and a three-dimensional en face view show the successful systolic anterior motion–related mitral regurgitation reduction by an XT clip implantation (asterisks) within an acceptable degree of the mean transmitral valve pressure gradient.

Alcohol septal ablation was performed using a 7 Fr guiding catheter via the right radial artery. Simultaneously, intraventricular pressure was monitored with a pressure wire (Abbott Vascular, CA, USA) using a 5 Fr guiding catheter via the left radial artery. Alcohol septal ablation targeting the septal perforators that potentially contributed the most to the LVOT obstruction was performed using a total absolute ethanol injection of 2.5 mL; however, this failed to sufficiently reduce the LVOT PG (maximum and mean LVOT PG, 81.6 and 35 mmHg, respectively) (*Figure 2A*and*B*). Moreover, this gradually led to interventricular septal oedema, which caused an acute deterioration of SAM-related MR from mild to massive at 6 h post-ASA (*Figure 2C*and*D*; see Supplementary material online, *Videos S1* and *S2*). A continuous infusion of landiolol, diltiazem, and single-dose methylprednisolone was ineffective in reducing SAM-related MR. The patient was haemodynamically unstable (systolic blood pressure of 50 mmHg), even under noradrenaline, and experienced deteriorating oxygen saturation (SpO_2_ 88%), even under invasive positive pressure ventilation support. Finally, venous-arterial extracorporeal membrane oxygenation (ECMO) and a percutaneous left ventricular assist device (Impella CP, Abiomed, MA, USA) were required for refractory pulmonary oedema caused by massive SAM-related MR. Transoesophageal echocardiography showed that SAM-related MR was caused by post-ASA morphological changes leading to a large coaptation gap (1.4 mm) of mitral leaflets with a pseudo-prolapse of the posterior mitral leaflet (PML) at a length of 10.2 mm (*Figure 3A*and*B*). The mitral valve area was relatively small (3.66 cm^2^) with a mean transmitral PG of 2.1 mmHg. Because the patient’s surgical risk was prohibitively high due to the sub-acute post-ASA phase and the mitral valve morphology was treatable by M-TEER, our heart team decided to perform MitraClip therapy.

During M-TEER, just before the clip insertion, we removed the Impella device to avoid these interferences. Thereafter, an XT clip (Abbott Vascular) was used to grasp the centre of the mitral valve (A2/P2), including the pre-existing elongated anterior mitral leaflet (AML) with a length of 29 mm; however, the mean transmitral valve-PG remarkably increased to 8 mmHg with mild LVOT obstruction. The XT clip was then laterally relocated to cover the pseudo-prolapsed PML, resulting in optimal MR reduction with an acceptable mean transmitral valve-PG (2.8 mmHg) (*Figure 3C*and*D*; see Supplementary material online, *Video S3*). This led to an improvement in the patient’s oxygen saturation level, and he was successfully weaned from ECMO on post-MitraClip Day 2. After that, he was treated with beta-blocker alone, resulting in moderate LVOT obstruction (LVOT PG of 29 mmHg) with mild SAM-related MR. He was finally transferred to another hospital to continue his rehabilitation.

## Discussion

This case demonstrated that a rare complication of acutely deteriorated SAM-related MR caused by interventricular septal oedematous protrusion following ASA could be successfully treated with MitraClip therapy, saving the patient from a catastrophically unstable condition.

The long-term outcomes after ASA and surgical myectomy, including all-cause mortality and sudden cardiac death, are reportedly similarly low. However, the frequency of re-intervention or permanent pacemaker implantation is higher in patients who undergo ASA rather than surgical myectomy, while periprocedural mortality tends to be higher with surgical myectomy than ASA.^7^ In the present case, SAM of the mitral valve was possibly caused by septal hypertrophy and elongated AML. Therefore, transcatheter septal reduction therapy by ASA was selected based on the patient’s desire for a less-invasive treatment. However, ASA led to cardiogenic shock with heart failure deterioration owing to acute septal oedema. This case demonstrated that a strengthened ‘Venturi effect’, caused by ASA-induced interventricular septal oedematous protrusion and pre-existing elongated AML, produces a large coaptation gap of mitral leaflets, resulting in SAM-related MR deterioration. Thus, pre-evaluation using transoesophageal echocardiography for the aetiology of LVOT obstruction and SAM-related MR is important in guiding optimal strategy. In cases with anatomical causes other than septal hypertrophy for LVOT obstruction and SAM-related MR, surgical interventions would be suitable.

The efficacy of M-TEER for SAM-related MR following various causes, including acute aortic dissection and surgical mitral annuloplasty, has been reported;^6,7^ however, its efficacy for failed ASA-induced SAM-related MR has never been reported. In our patient case, septal morphological changes led to a large leaflet coaptation gap, and MitraClip therapy was deemed a suitable treatment. A small mitral valve area is highly risky for iatrogenic mitral stenosis following MitraClip therapy.^8^ In this patient, although MitraClip therapy grasping of elongated AML was assumed to be effective for LVOT obstruction and SAM-related MR, an unacceptable degree of iatrogenic mitral stenosis (>5 mmHg) occurred owing to the small mitral valve area (<4 cm^2^) consisting of elongated mitral leaflets. Based on the findings, MitraClip therapy mainly grasping of the pseudo-prolapse of PML was performed in the present case.

MitraClip therapy is a feasible bailout treatment for SAM-related MR following ASA in patients with an acceptable severity of iatrogenic mitral stenosis post-MitraClip implantation.

## Supplementary Material

ytad599_Supplementary_Data

## Data Availability

The data included in this study are available in the article and its Supplementary material online.

## Lead author biography

Dr Shinsuke Nakano received his licence to practice medicine at Miyazaki University. He currently works as a physician in the field of interventional cardiology at the Hyogo Prefectural Harima-Himeji General Medical Center, Himeji, Japan. His main area of research is intravascular coronary imaging and endovascular treatment.

## Supplementary material

**Consent:** Informed consent for the publication was obtained from the patient per the COPE guidelines.

**Funding:** None declared.
